# Metabolomic Profiling and In Vivo Antiepileptic Effect of *Zygophyllum album* Aerial Parts and Roots Crude Extracts against Pentylenetetrazole-Induced Kindling in Mice

**DOI:** 10.3390/metabo14060316

**Published:** 2024-05-30

**Authors:** Asmaa R. Abdel-Hamed, Alaa S. Wahba, Dina M. Khodeer, Maged S. Abdel-Kader, Jihan M. Badr, Sebaey Mahgoub, Dina M. Hal

**Affiliations:** 1 Department of Biochemistry, Faculty of Pharmacy, Suez Canal University, Ismailia 41522, Egypt; asmaa.ramdan@pharm.suez.edu.eg (A.R.A.-H.); alaa.samir@pharm.suez.edu.eg (A.S.W.); 2 Department of Pharmacology and Toxicology, Faculty of Pharmacy, Suez Canal University, Ismailia 41522, Egypt; dina_khoudaer@pharm.suez.edu.eg; 3 Department of Pharmacognosy, College of Pharmacy, Prince Sattam Bin Abdulaziz University, Al-Kharj 11942, Saudi Arabia; 4 Department of Pharmacognosy, Faculty of Pharmacy, Alexandria University, Alexandria 21215, Egypt; 5 Department of Pharmacognosy, Faculty of Pharmacy, Suez Canal University, Ismailia 41522, Egypt; gehan_ibrahim@pharm.suez.edu.eg (J.M.B.); dina_hal@pharm.suez.edu.eg (D.M.H.); 6 Food Analysis Laboratory, Ministry of Health, Zagazig 44511, Egypt; dr_semahgoub@yahoo.com

**Keywords:** metabolomic profiling, LC-ESI-TOF-MS/MS, *Zygophyllum album*, PTZ, oxidative stress, NLRP3 inflammasome, apoptosis

## Abstract

The chemical profiles of both *Zygophyllum album* (*Z. album*) aerial parts and roots extracts were evaluated with LC-ESI-TOF-MS/MS analysis. Twenty-four compounds were detected. Among them, some are detected in both the aerial parts and the roots extracts, and others were detected in the aerial parts only. The detected compounds were mainly flavonoids, phenolic compounds, triterpenes and other miscellaneous compounds. Such compounds contribute to the diverse pharmacological activities elicited by the *Z. album* species. This study aimed to elucidate the antiepileptic effect of *Z. album* aerial parts and roots crude extracts against pentylenetetrazole (PTZ)-induced kindling in mice. Male albino mice were divided into four groups, eight animals each. All groups, except the control group, were kindled with PTZ (35 mg/kg i.p.), once every alternate day for a total of 15 injections. One group was left untreated (PTZ group). The remaining two groups were treated prior to PTZ injection with either *Z. album* aerial parts or roots crude extract (400 mg/kg, orally). Pretreatment with either extract significantly reduced the seizure scores, partially reversed the histological changes in the cerebral cortex and exerted antioxidant/anti-inflammatory efficacy evinced by elevated hippocampal total antioxidant capacity and SOD and catalase activities, parallel to the decrement in MDA content, iNOS activity and the TXNIB/NLRP3 axis with a subsequent decrease in caspase 1 activation and a release of IL-1β and IL-18. Moreover, both *Z. album* extracts suppressed neuronal apoptosis via upregulating Bcl-2 expression and downregulating that of Bax, indicating their neuroprotective and antiepileptic potential. Importantly, the aerial parts extract elicited much more antiepileptic potential than the roots extract did.

## 1. Introduction

Natural products have been used traditionally to cure a wide variety of illnesses for thousands of years. It has been estimated that natural ingredients represent about half of currently used pharmaceuticals [1,2,3]. Zygophyllaceae is one of the biggest families, with approximately 25 genera and 240 species [4]. *Zygophyllum*, a genus that belongs to the family Zygophyllaceae, is of common occurrence in Egypt. It has been used traditionally for curing various diseases such as hypertension, asthma, gout and diabetes [4,5].

Previous studies reported diversity of the chemical constituents and the interesting biological activities of *Zygophyllum album.* Our previous investigation of *Z. album* aerial parts has led to the isolation and identification of a number of compounds, such as *β*-amyrin, ursolic acid, caffeic acid, kaempferol, quercetin, rutin and a new saponin assigned the name Zygo-albuside A [6]. Additionally, investigation of root extracts of the same plant has led to isolation and chemical identification of a new compound named Zygo-albuside D along with the known compounds (3-O-[*β*-D-quinovopyranosyl]-quinovic acid) and catechin [3]. These compounds were isolated using different chromatographic techniques and identified with 1D and 2D NMR and HRMS spectral data. *Z. album* was reported to have antioxidant [6], antiobesity, antiacetylcholinesterase [7], antihypertensive, antidiabetic [8], and anticancer activities [9].

Epilepsy is a chronic neurological disease characterized by frequent seizures. Epileptogenesis has been reported to be elicited by either genetic or acquired factors that increase one’s susceptibility to seizures [10]. Epileptic seizures are associated with various insults including oxidative stress, neuronal cell damage, abnormal neurogenesis, deregulated apoptosis and autophagy subsequently leading to cognitive impairment and neuroinflammation [11,12,13]. Interestingly, a principal platform of the inflammatory signaling pathway that senses both pathogenic microorganisms and sterile stressors is the inflammasome. The latter is a multi-protein complex formed from the assembly of a sensor protein (pattern recognition receptor (PRR) containing a pyrin and/or a caspase recruitment and activation domain), an adaptor protein (apoptosis-associated speck-like protein (ASC)) and a caspase-1 effector protein [14]. The most classic inflammasome is nucleotide oligomerization domain-like receptor protein 3 (NLRP3) which consists of NLRP3 as the sensor protein. Stimulation with danger signals enhances recruitment and assembly of NLRP3 inflammasome components eventually leading to pro-caspase-1 cleavage into its active form, which in turn promotes the activation and secretion of inflammatory cytokines including interleukin (IL)-1β and IL-18. NLRP3 inflammasome has been reported to be involved in many neurological disorders such as Alzheimer’s disease, cerebrovascular disease and epilepsy [10].

The available antiepileptic drugs have been reported to be associated with serious side effects such as agranulocytosis, teratogenicity, addiction and tolerance. Additionally, the lack of good control and subsequent progression to drug-resistant epilepsy were recorded in almost one third of patients. In this regard, studies revealed that the children exposed to valproic acid demonstrated a high rate of facial dysmorphism and dental anomalies [15]. Moreover, a large percentage of patients with epilepsy who take antiepileptic drugs have suffered from bone abnormalities. Drugs such as benzodiazepines, carbamazepine, phenytoin, phenobarbital and valproic acid cause induction of CYP450 isoenzymes. This in turn can lead to vitamin D deficiency, hypocalcemia, increased risk for fracture and altered bone turnover, causing impaired bone mineral density [16]. Therefore, there is an urgent need for development of novel antiepileptic drugs that can mitigate epileptogenesis effectively and safely. Natural anti-inflammatory therapies have been suggested as promising alternatives for preventing and treating epileptic seizures by virtue of their multi-targeted effects [10,11].

This study aimed to investigate both *Z. album* aerial parts and roots crude extracts, using the LC-ESI-TOF-MS/MS analysis technique, and the potential antiepileptic effect of *Z. album* against pentylenetetrazole (PTZ)-induced kindling in mice is evaluated. A substantial goal is to establish a comparative study between the *Z. album* aerial parts and roots crude extracts. Finally, the underlying mechanisms of action (in vitro and in vivo) were assessed with attention being paid to thioredoxin (TRX)-interacting protein (TXNIP)/NLRP3 inflammasome signaling as a respectable epileptic therapeutic target.

## 2. Materials and Methods

### 2.1. Plant Material

*Z. album* was collected from Marsa Matrouh at the Northern Coast of the Mediterranean Sea in Egypt during May 2019. It was identified at the Faculty of Science, Alexandria University. A voucher specimen was kept under registration number ZA-2019 in the herbarium of the Pharmacognosy Department, Faculty of Pharmacy, Suez Canal University, Ismailia, Egypt. The plant was air-dried at shade and room temperature (around 25 °C) for 2 weeks, the aerial parts were separated from the roots then each were chopped separately into small pieces. A weight of 1.8 kg of *Z. album* was extracted with methanol (3 × 2 L) at room temperature. The combined aerial extracts were concentrated under reduced pressure to yield brownish-green viscous crude extract (50 g), and about 900 g of *Z. album* roots were extracted with methanol (3 × 2 L) at 25 °C. The combined roots extracts were concentrated under reduced pressure to afford 30 g of brownish-green crude extract.

### 2.2. Metabolomic Profiling with LC/MS/MS

High-resolution LC-ESI-TOF-tandem mass spectrometric analysis was executed as previously mentioned in [17]. For the *Zygophyllum album* aerial and roots extracts, 50 mg of each extract were dissolved in 1 mL of solvent mixture composed of water:methanol:acetonitrile (50:25:25, *v*/*v*). The extract solution was sonicated (10 min) and then centrifuged at 10,000 rpm (10 min) to ensure complete solubility. An aliquot (50 µL) of the prepared solution was withdrawn and further diluted with the solvent mixture.

Finally, 2.5 µg/µL of the extract was prepared, of which 10 µL was injected in both negative and positive modes. For confidence assurance in our experiment, blanks were also analyzed.

The chromatographic separation was performed using a 28 min gradient elution program with a constant flow rate of 0.3 mL/min. In the positive mode, mobile phase A consisted of 5 mM ammonium formate buffer in 1% methanol (pH = 3.0), while mobile phase B was acetonitrile. Conversely, for the negative mode, we employed 5 mM ammonium formate in 1% methanol (pH 8.0) as mobile phase A, along with acetonitrile as mobile phase B.

The UHPLC separation was achieved using an ExionLC system (AB Sciex, Framingham, MA, USA) with a 2.5 µm, 2.1 × 150 mm XSelect HSS T3 column (Waters Corporation, Milford, MA, USA), Phenomenex^®^ in-line filter disks (0.5 µm × 3.0 mm) and an autosampler system. The gradient elution followed these steps: 0% B for 1.0 min, 0–90% B in 20 min, 90% for 4.0 min, 90–0% B in 1.0 min and, finally, re-equilibration with 0% B for 3.0 min.

For mass spectrometry, this UHPLC was attached to a Triple TOF™ 5600^+^ system equipped with a Duo-Spray source operating in the electrospray ionization (ESI) mode (AB SCIEX, Concord, ON, Canada). Sprayer capillary and declustering potential voltages were set to 4500 and 80 eV in the positive mode and −4500 and −80 V for the negative mode using a source temperature of 600 °C. The curtain gas was 25 psi, and gas 1 and gas 2 were 40 psi. The TripleTOF5600^+^ operated using an information-dependent acquisition (IDA) protocol. The collision energy was 35 V (positive mode) and −35 V (negative mode) with CE spreading 20 V and ion tolerance of 10 ppm.

The information-dependent acquisition (IDA) method was employed to simultaneously collect full-scan MS and MS/MS data. This method involved acquiring high-resolution survey spectra across the mass range from 50 to 1100 *m/z*. During operation, the mass spectrometer followed a pattern where a 50 ms survey scan was detected. Subsequently, the top 15 intense ions were selected for acquiring MS/MS fragmentation spectra after each scan.

MSDIAL3.52 was utilized for data processing. Master view software was employed for the peak extraction from the total ion chromatogram (TIC) according to the criteria reported previously by [17]. The compounds were identified with accurate mass estimations, MS/MS transitions and the comparison of their retention time to those reported in the literature and mass spectral databases for LC/MS-based metabolomic analysis.

### 2.3. In Vivo Study of Z. album

#### 2.3.1. Drugs and Chemicals

Pentylenetetrazole (PTZ) was purchased from Sigma Aldrich (St. Louis, MI, USA) with 0.9% sterile saline being utilized for PTZ dissolution.

#### 2.3.2. Experimental Animals and Study Protocol

Animal handling and experimental protocols were approved by the research ethics committee at the Faculty of Pharmacy, Suez Canal University (Ethics code. 202307RA2) and followed the Guide for the Care and Use of Laboratory Animals (8th edition, National Academies Press) [18]. 

Thirty-two Swiss male albino mice, delivered from the Serum and Vaccine authority (Cairo, Egypt), were used in this study with weights ranging from 20 to 25 g. Mice were housed in stainless steel cages in groups of 8 animals per cage. Housing was under a normal light/dark cycle with controlled room temperature (25 ± 1 °C), relative humidity (55–65%) and free access to food and water. A one-week adaptation period was allowed before starting the experiment.

Animals were randomly allocated into four experimental groups (eight mice per group). The mice in the first group received 1% DMSO/distilled water orally and sterile saline (0.9% NaCl) intraperitoneally (i.p.) and served as the normal control (NC) group. Kindling was induced in the remaining three groups via injecting animals with a sub-convulsive dose of PTZ (35 mg/Kg, i.p.) twice a week for five weeks to reach a total of 15 injections. Kindling was achieved after 14 ± 1 PTZ injections or when the mouse showed a seizure score of 4.5 or 5 in three consecutive occasions [19]. Mice in the PTZ group were left untreated, while mice in the aerial extract and root extract groups received a dose of 400 mg/Kg, orally (p.o.) of either *Z. album* aerial extract or root extract resuspended in 1% DMSO/distilled water 30 min before each PTZ injection. The dose of *Z. album* extract from both aerial parts and roots was selected according to previous studies that utilized the same dose of the extract and reported potential cardioprotective and antihyperlipidemic effects [7,20]. The animals received equal doses from both extracts rather than doses calculated based on the total phenolic content due to presence of other metabolites in both extracts that may possess potent antiepileptic activity. Interestingly, two of these metabolites were isolated for the first time from a natural source [3,6].

#### 2.3.3. Assessment of Seizure Activity in PTZ-Kindled Mice

For all mice, convulsive behavior was observed in a blind manner for 30 min after each PTZ injection. The seizure score was classified into the following stages, as described by Fischer and Kittner: 0, no seizure; 0.5, weak nodding; 1, ear, face and eyelid spasms; 2, myoclonic jerks, no rearing; 2.5, rapid clonic forelimb seizures, partial rearing; 3, severe bilateral forelimb clonuses, complete rearing; 3.5, rearing and falling with forceful forelimb clonus; 4, generalized clonic seizures with jumping or episodes of rearing-falling down; 4.5, generalized clonic–tonic seizures with loss of righting reflex; 5, generalized clonic–tonic seizures and status epilepticus. For each group, the mean of the reported seizure scores over PTZ fifteen injections was calculated and used for comparison [21].

#### 2.3.4. Tissue Sampling

Following convulsive behavioral assessment after the last PTZ injection, mice were injected with ketamine (10 mg/kg, i.p.) and euthanized via decapitation with the brain tissues being instantly excised. The whole cerebrum in both the right and left hemisphere without cerebellum and brain stem was weighed, cut sagittally into left and right hemispheres and blotted dry on tissue paper after rinsing in ice-cold physiological saline. The right hemisphere was dipped in liquid nitrogen (−170 °C, provided from the Directorate of Veterinary Medicine, Zagazig, Egypt) for 5 min and then kept frozen at −80 °C for further biochemical and RT-PCR analysis. The left hemisphere was fixed in 10% neutral buffered formaldehyde (Sigma Aldrich Chemical Company, St. Louis, MO, USA, Cat. No. HT501128) for histopathological examination.

#### 2.3.5. Determination of Oxidative Stress Markers (iNOS, TAC, MDA, SOD and Catalase)

Following the manufacturer’s instructions, iNOS and TAC were measured in the brain tissue of the groups under study using the mouse inducible nitric oxide synthase (iNOS) ELISA Kit (MyBioSource, San Diego, CA, USA, Cat. No. MBS261100) and mouse total antioxidant capacity ELISA Kit (MyBioSource, San Diego, CA, USA, Cat. No. MBS733680) [22,23], respectively.

The brain tissue’s levels of malondialdehyde (MDA), superoxide dismutase (SOD) and catalase were assessed using Biodiagnostic colorimetric assay kits (Dokki, Giza, Egypt, Cat. No. MD 2529 for MDA, SD 2521 for SOD, and CA 2517 for catalase). The manufacturer’s instructions were followed for every procedure [23].

#### 2.3.6. Determination of the Inflammatory Markers (IL-1β and IL-18)

Levels of interleukin (IL)1β and IL-18 were measured in the brain tissue of the study groups using mouse-specific ELISA kits purchased from MyBioSource (San Diego, CA, USA, Cat. No. MBS701092 and MBS9135813, respectively) and the manufacturer’s instructions were followed [23,24].

#### 2.3.7. Quantitative RT-PCR Analysis for Determination of Inflammatory Biomarkers

By using the ABT Total RNA Mini Extraction Kit (Applied Biotechnology, Ismailia, Egypt, Cat. No. ABT001), RNA was extracted from the brain tissue of the study groups according to the protocol supplied by the manufacturer. RNA concentration and purity were measured spectrophotometrically using a NanoDrop 1000 spectrophotometer (NanoDrop Tech, Wilmington, DE, USA). 

The expression of *NLRP3*, *TXNIP* and *caspase-1* genes were assessed using the GoTaq^®^ 1-Step RT-qPCR System (Promega, Madison, WI, USA, Cat. No. A6020) and glyceraldehyde 3-phosphate dehydrogenase (*GAPDH*) as a reference gene. Table 1 shows the primer sequence that was employed as well as the annealing temperature for each primer. In a final volume of 20 μL, the reaction was conducted using the following components: 4 μL RNA template, 0.4 μL GoScript™ RT mix for 1-step RT-qPCR, 1 μL of each forward and reverse primer, 10 μL GoTaq^®^ qPCR master mix, 0.31 μL additional CXR reference dye and 3.29 μL nuclease-free water. Cycle conditions included 15 min of reverse transcription at 37 °C, 10 min of reverse transcriptase enzyme inactivation at 95 °C, 40 cycles of denaturation at 95 °C for 10 s, annealing for 30 s and extension at 72 °C for 30 s. All real-time PCR reactions were performed in a StepOnePlus™ Real-Time PCR thermal cycling instrument (Applied Biosystems, Waltham, MA, USA). The 2^−ΔΔCT^ method was utilized to ascertain the relative expression of the evaluated genes [25].

#### 2.3.8. Histopathological Study of Brain Tissue Sections in Different Groups

Brain tissue sections were evaluated for the neurons and glial tissue in the cerebral cortex and for any pathological changes. Neurons were counted in 3 different high-power fields (hpf) (400×). The severity of microscopic lesions observed were graded based on the degree and extent of tissue damage using a four-point scale: absent (grade 0), no lesions detected; minimal (grade 1), lesions involved less than 10% of the tissue section; mild (grade 2), lesions involved 11–40% of the tissue section and moderate (grade 3), lesions involved 41–80% of the tissue section, modified from Jokinen et al. [30]. Neuronal tissues were examined for the following pathological changes: red neurons, perineuronal edema, inflammatory infiltrate, neuronal pyknosis, necrotic neurons, areas of necrosis and areas of reactive gliosis.

#### 2.3.9. Immunohistochemical Staining and Determination of Apoptotic Markers Immunoexpression (Bcl-2 and Bax)

Sections from the selected paraffin blocks were cut into 4 μm thick sections for immunohistochemical (IHC) staining. Slides were prepared and incubated with primary anti-Bcl-2 antibody and anti-Bax antibody were obtained from Abcam (Cambridge, UK). This was followed by incubation with the appropriate secondary antibody. All slides were counterstained with hematoxylin for 30 s prior to dehydration and mounting. Neuronal cytoplasmic/nuclear reaction to antibody was considered positive. Semi-quantitative analysis of stained tissue sections was performed following the Allred scoring system [31]. Photographing was performed under a light microscope and photographs were later employed to measure the immunoreactivity to Bax and Bcl-2 using an image analysis system “ImageJ 1.45F” (National Institute of Health, Bethesda, MD, USA). 

#### 2.3.10. Statistical Analysis

The statistical tests were carried out using GraphPad Prism version 7.0.1 (GraphPad Software, Inc., San Diego, CA, USA). Data were presented as mean ± standard deviations (SD). One-way ANOVA followed by Tukey’s post hoc test were used to compare means of different groups. *p* values less than 0.05 were considered statistically significant.

## 3. Results and Discussion

### 3.1. LC-ESI-TOF-MS/MS Analysis of Zygophyllum album

Since *Zygophyllum album* was reported to be a rich source of miscellaneous active compounds, aerial parts and roots crude extracts were evaluated with the LC-ESI-TOF-MS/MS analysis technique (AB SCIEX, Concord, ON, Canada) in order to fully understand the chemical diversity of its phytoconstituents including flavonoids and other metabolites. Data are represented in Table 2 and Table 3 and the LC-ESI-TOF-MS/MS profile is shown in (Supplementary Materials Figures S1–S4). Comparing the chromatographic behaviour, *m/z* values in the total ion chromatogram (TIC) and base peak chromatogram (BPC) profiles, as well as the fragmentation pattern, with those described in the literature, allowed for the possible identification of the individual components. The mass accuracy was calculated as follows:

[Measured mass-expected mass/expected mass] × 10^6^ and expressed in parts per million (ppm) error [32]. 

**Figure 1:**
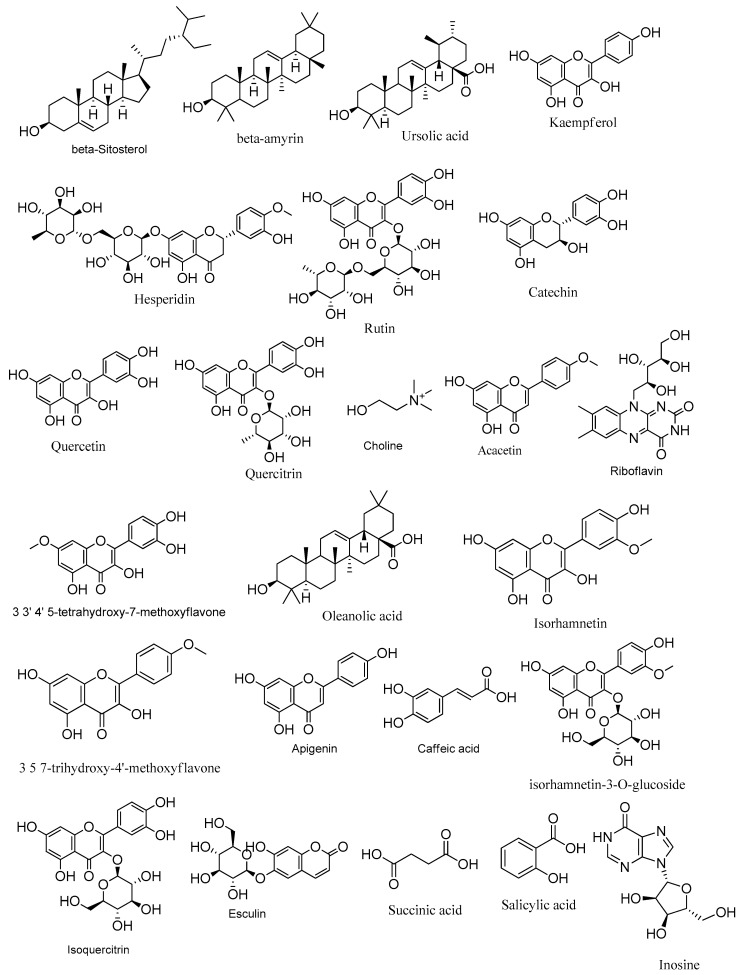
Metabolites identified in *Zygophyllum album* crude aerial and roots extracts using LC-ESI/TOF/MS/MS.

More precisely, 24 hits were identified in *Z. album* (Table 2 and Table 3, Figure 1) belonging to different chemical classes; mainly flavonoids. Thirteen flavonoids have been detected in both *Z. album* crude aerial parts and roots extracts among which rutin, quercetin, kaempferol, isorhamnetin-3-*O*-glucoside, isorhamnetin and isoquercetin were previously isolated from the aerial parts crude extract of *Z. album* [5,6,33]. These compounds are reported here for the first time in the roots crude extract. The catechin flavonoid was previously isolated from the roots extract [3] and detected in this study for the first time in the aerial parts extract. In addition, acacetin, quercitrin, 3, 5, 7-trihydroxy-4′-methoxy flavone (Kaempferide) and hesperidin are reported in this investigation for the first time only in the aerial parts extract. 

Apigenin and 3,3′,4′,5-tetrahydroxy-7-methoxyflavone are reported in this investigation for the first time in both the aerial parts and roots crude extracts. Additionally, three triterpenes, ursolic acid, oleanolic acid and *β*-amyrin that have been previously isolated from the aerial parts extract [6] are reported in this study in both the aerial parts and the roots extract. Moreover, the aerial parts extract exhibited the presence of caffeic acid, succinic acid, (-)-riboflavin and esculin while *β*-Sitosterol and choline were shown in both extracts.

**Table 2:** Metabolites identified in *Zygophyllum album* crude extract of both aerial and roots parts using LC-ESI/TOF/MS/MS (Positive Mode, 5 mM ammonium formate buffer in 1% methanol, pH 3: Acetonitrile).

No.	Ret. Time (min)	Measured *m/z*	*Calculated* *m/z*	Mass Error (ppm)	Adduct	Molecular Formula	MS/MS Spectrum	Deduced Name	Ref.	Plant Part
1	26.52	415.3951	415.3940	2.65	[M + H]^+^	C_29_H_50_O	255, 147	*β*-Sitosterol	[34,35]	Roots
		415.3935		−1.20						Aerial
2	22.88	427.3952	427.3940	2.81	[M + H]^+^	C_30_H_50_O	177, 259, 299	*β*-amyrin	[36]	Roots
		427.3928		−2.81						Aerial
3	22.58	457.3687	457.3682	1.09	[M + H]^+^	C_30_H_48_O_3_	203, 161, 95	Ursolic acid	[37,38]	Roots
		457.3674		−1.75						Aerial
4	9.99	287.0562	287.0556	2.09	[M + H]^+^	C_15_H_10_O_6_	241, 223	Kaempferol	[39,40]	Roots
		287.0543		−4.53						Aerial
5	6.41	611.1993	611.1976	2.78	[M + H]^+^	C_28_H_34_O_15_	611	Hesperidin	[41]	Aerial only
6	6.39	611.1582	611.1612	−4.91	[M + H]^+^	C_27_H_30_O_16_	303, 609	Rutin	[42,43]	Roots
		611.1588		−3.93						Aerial
7	4.62	291.0857	291.0869	−4.12	[M + H]^+^	C_15_H_14_O_6_	205, 179	Catechin	[44]	Roots
		291.0863		−2.06						Aerial
8	9.51	303.0520	303.0505	4.95	[M + H]^+^	C_15_H_9_O_7_	68, 121	Quercetin	[45]	Roots
		303.0491		−4.62						Aerial
9	7.23	449.1064	449.1084	−4.45	[M + H]^+^	C_21_H_20_O_11_	254, 346	Quercitrin	[46]	Aerial only
10	1.19	104.1071	104.1070	0.96	[M + H]^+^	C_5_H_14_NO	104, 60	Choline	[47]	Roots
		104.1066		−3.84						Aerial
11	14.16	285.0762	285.0763	−0.35	[M + H]^+^	C_16_H_12_O_5_	285, 193, 153	Acacetin	[48]	Aerial only
12	14.38	377.1446	377.1461	−3.98	[M + H]^+^	C_17_H_20_N_4_O_6_	359, 341	(-)-Riboflavin	[49]	Aerial only
13	8.71	317.0657	317.0661	−1.26	[M + H]^+^	C_16_H_12_O_7_	302, 224	3,3′,4′,5-tetrahydroxy-7-methoxy flavone(Rhamnetin)	[50]	Roots
		317.0648		−4.10						Aerial
14	22.53	457.3659	457.3682	−5.03	[M + H]^+^	C_30_H_48_O_3_	457, 393	Oleanolic acid	[51]	Roots
		457.3674		−1.75						Aerial
15	7.48	317.0647	317.0661	−4.42	[M + H]^+^	C_16_H_12_O_7_	300, 151	Isorhamnetin	[52,53]	Roots
		317.0643		−5.67						Aerial
16	9.59	301.0701	301.0712	−3.65	[M + H]^+^	C_16_H_12_O_6_	269, 349	3,5,7-trihydroxy-4′-methoxyfla-vone (Kaempferide)	[54]	Aerialonly

**Table 3:** Metabolites identified in *Zygophyllum album* crude extract of both aerial and roots parts using LC-ESI/TOF/MS/MS (Negative Mode, 5 mM ammonium formate buffer in 1% methanol, pH 8: Acetonitrile).

No.	Ret. Time (min)	Measured *m/z*	*Calculated* *m/z*	Mass Error (ppm)	Adduct	Molecular Formula	MS/MS Spectrum	Deduced Name	Ref.	Plant Part
1	10.32	269.0439	269.0450	−4.09	[M − H]^−^	C_15_H_10_O_5_	269	Apigenin	[43]	Roots
		269.0459		3.35						Aerial
2	1.33	179.0343	179.0344	−0.56	[M − H]^−^	C_9_H_8_O_4_	180, 161	Caffeic acid	[43]	Aerial only
3	7.27	477.1031	477.1033	−0.42	[M − H]^−^	C_22_H_22_O_12_	477, 314, 285, 271, 243	isorhamnetin-3-*O*-glucoside	[55,56,57]	Roots
		477.1032		−0.21						Aerial
4	6.22	463.0879	463.0877	0.43	[M − H]^−^	C_21_H_20_O_12_	343, 303	Isoquercitrin	[58]	Roots
		463.0879		0.43						Aerial
5	7.15	339.0699	339.0716	−5.01	[M − H]^−^	C_15_H_16_O_9_	133, 148	Esculin	[59]	Aerial only
6	1.08	117.0192	117.0188	3.42	[M − H]^−^	C_4_H_6_O_4_	99	Succinic acid	[60]	Aerial only
7	1.31	137.0242	137.0239	2.19	[M − H]^−^	C_7_H_6_O_3_	75, 93	Salicylic acid	[61]	Roots
		137.0241		1.46						Aerial
8	1.13	267.0734	267.0729	1.87	[M − H]^−^	C_10_H_12_N_4_O_5_	267, 113, 92, 89, 71, 59	Inosine	[62]	Roots
		267.0716		4.87						Aerial

### 3.2. The Antiepileptic Effect of Z. album Aerial Parts and Roots Crude Extracts against Pentylenetetrazole (PTZ)-Induced Kindling in Mice

**Figure 2:**
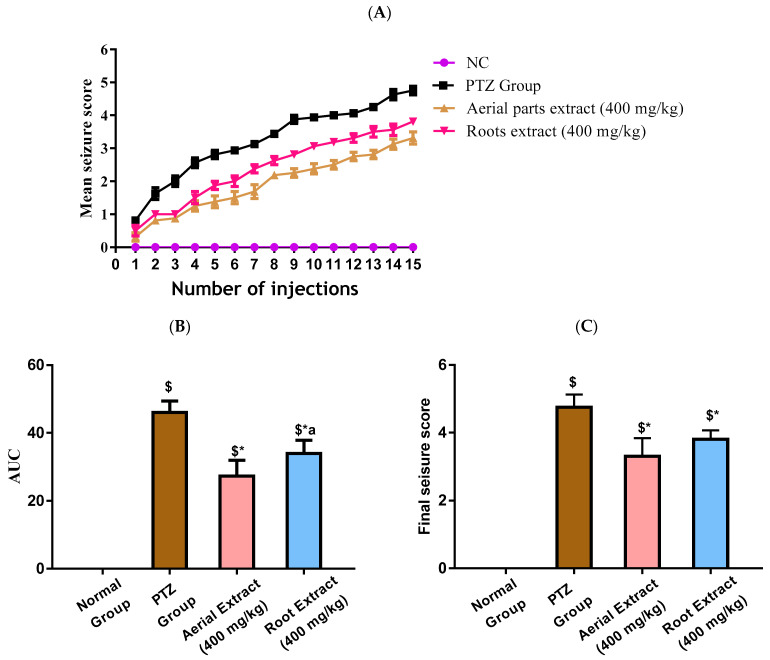
Induction of kindling was achieved following fifteen PTZ injections (35 mg/Kg, i.p., three times a week for five weeks) with the seizure scores being assessed according to the Fischer and Kittner scoring scale. The effect of *Z. album* aerial parts and roots crude extracts on (**A**) mean seizure scores over the fifteen PTZ injections, (**B**) the AUC and (**C**) the final seizure score of the experimental mice. PTZ = pentylenetetrazole and AUC = area under the curve. Data are expressed as mean ± SD. Analysis was performed using one-way ANOVA followed by Tukey’s post hoc test (*n* = 8). ^$^ significantly different vs. the normal control group; * significantly different vs. the PTZ group; ^a^ significantly different vs. the aerial extract (400 mg/kg) group. Differences were considered significant at *p* < 0.01.

In the current study, kindling in mice was induced following fifteen PTZ injections, as shown in Figure 2A. A progressive increase in the seizure scores was observed in PTZ mice with elevated seizure scores at all injection days and consequently increased AUC (*p* < 0.001), as compared to the control group. Treatment with either aerial parts or roots crude extracts resulted in decreased seizure scores at all injection days, corroborated by a reduced AUC compared to the PTZ mice (*p* < 0.001) with the greater reduction in the AUC being observed in the aerial parts crude extracts (Figure 2B). The final seizure score in each experimental group was compared. The final seizure score exhibited by PTZ mice was significantly higher than that exhibited by the control mice (4.63 ± 0.52 versus 0 ± 0, *p* < 0.001, Figure 2C). Treatment with aerial parts and roots crude extracts significantly decreased the final seizure score compared to the PTZ group. Of note, the score recorded in mice in either treatment group was not significantly different.

Importantly, all experimental mice in the PTZ group (stage 4 and 5 seizures), while 62.5% of mice in root extract group and only 25% of mice in aerial extract group, were fully kindled. There was no appearance of stage 5 seizures in the treatment groups.

**Figure 3:**
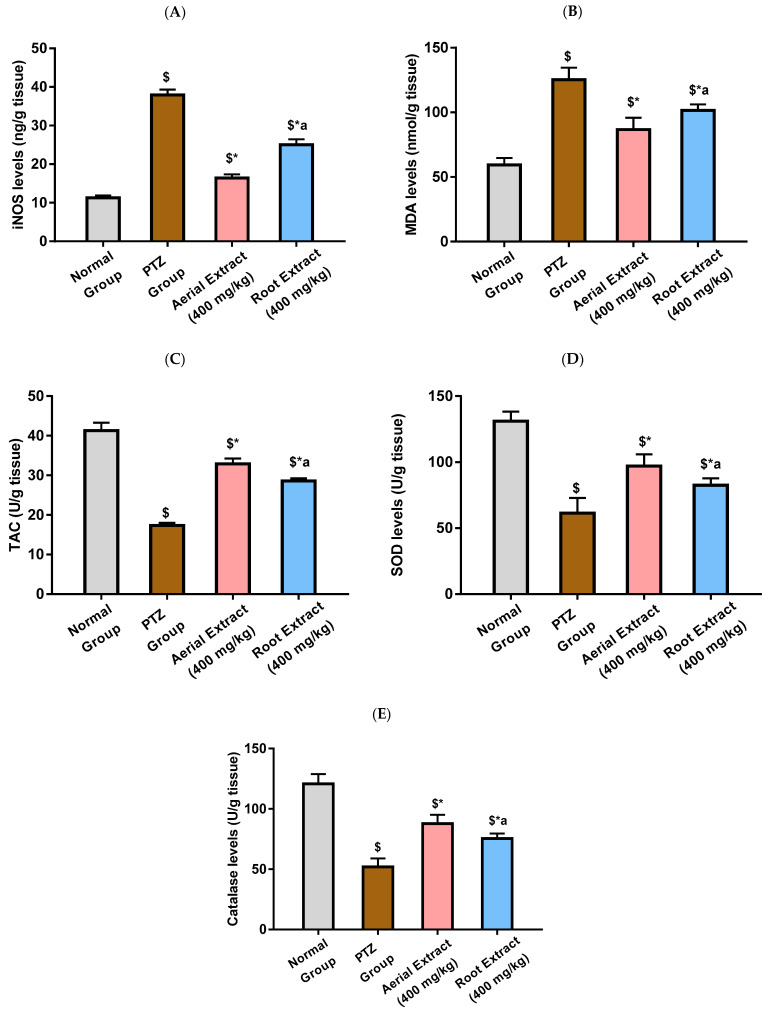
The effect of *Z. album* aerial parts and roots crude extracts on the levels of (**A**) iNOS, (**B**) MDA, (**C**) TAC, (**D**) SOD and (**E**) catalase. iNOS = inducible nitric oxide synthase; MDA = malondialdehyde and TAC = total antioxidant capacity. Data are expressed as mean ± SD. Analysis was performed using one-way ANOVA followed by Tukey’s post hoc test (*n* = 8). ^$^ significantly different vs. the normal control group; * significantly different vs. the PTZ group; ^a^ significantly different vs. the aerial extract (400 mg/kg) group. Differences were considered significant at *p* < 0.01.

The anti-inflammatory and antioxidant effect of *Z. album* aerial parts and roots crude extracts were investigated. Figure 3 reveals that PTZ administration was associated with significant elevation in the levels of the ROS-generating inflammatory oxidative enzyme, iNOS, and the lipid peroxidation marker, MDA, as well as a significant reduction in total antioxidant capacity and the levels of SOD and catalase, as compared with the normal group (*p* < 0.001). A significant decline in iNOS and MDA levels, as well as a significant increase in the total antioxidant capacity and SOD and catalase levels in the brain tissue, were observed in mice treated with *Z. album* aerial parts and roots crude extracts relative to the PTZ control group (*p* < 0.001). The most significant improvement in such parameters was exhibited by the group treated with *Z. album* aerial parts extract (*p* < 0.001).

NLRP3 inflammasome activation with subsequent caspase-1 cleavage and production of IL-1β and IL-18 has been reported to play an essential role in inflammatory and neurological diseases such as epilepsy. TXNIP is an essential intermediate that bridges redox signals with NLRP3 inflammasome activation [10,63].

**Figure 4:**
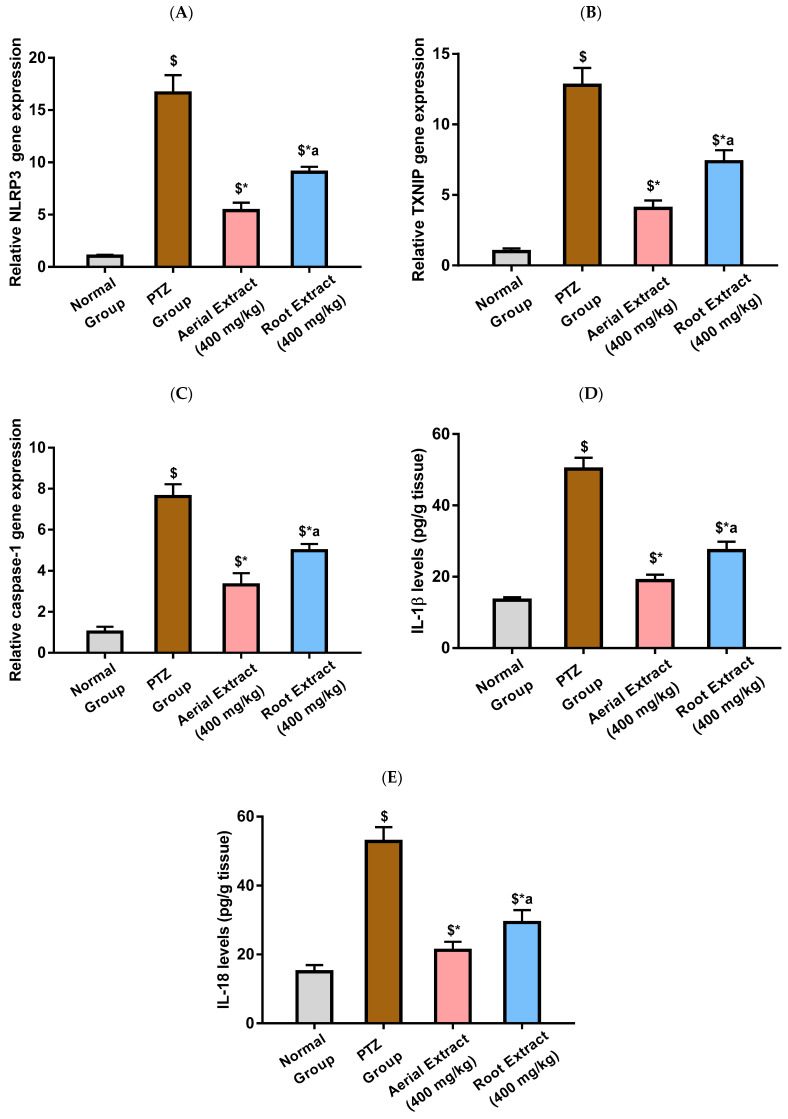
The effect of *Z. album* aerial parts and roots crude extracts on the relative gene expression of (**A**) *NLRP3*, (**B**) *TXNIP* and (**C**) *caspase-1* and levels of (**D**) IL-1β and (**E**) IL-18. *NLRP3* = nucleotide oligomerization domain-like receptor protein 3; *TXNIP* = Thioredoxin-interacting protein; IL-1β = interleukin-1 beta and IL-18 = interleukin-18. Data are expressed as mean ± SD. Analysis was performed using one-way ANOVA followed by Tukey’s post hoc test (*n* = 8). ^$^ significantly different vs. the normal control group; * significantly different vs. the PTZ group; ^a^ significantly different vs. the aerial extract (400 mg/kg) group. Differences were considered significant at *p <* 0.001.

As depicted in Figure 4A,E, the PTZ group showed a significantly activated NLRP3 inflammasome pathway, evinced by the 16.65-, 12.77- and 7.62-fold upregulation in *NLRP3*, *TXNIP* and *caspase-1* relative gene expression, respectively, as compared to the normal group (*p* < 0.001). It was in the same context that IL-1β and IL-18 levels were significantly elevated following PTZ injection by 2.74- and 2.53-fold, respectively, as compared to the normal group (*p* < 0.001). PTZ-induced upregulated expression of *NLRP3*, *TXNIP* and *caspase-1* and elevated levels of IL-1β and IL-18 were significantly reduced upon treatment with either *Z. album* aerial parts or roots crude extracts, as compared to the PTZ group (*p* < 0.001), pointing to their potential to inhibit NLRP3 inflammasome activation. Importantly, the inhibitory effect of *Z. album* aerial parts extract on NLRP3 inflammasome activation has been shown to be significantly superior to that of the roots crude extract (*p* < 0.001).

**Figure 5:**
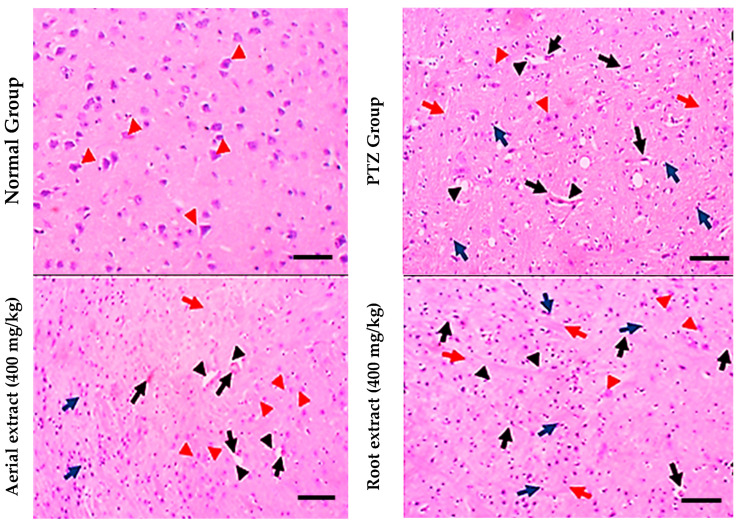
Histopathological stained brain sections. Normal group showed uniform neuronal tissue with many viable uniform neurons (red arrowheads). In the PTZ group, few viable neurons are seen (red arrowheads) with many red neurons (black arrows), perineuronal edema (black arrowheads) and gliosis (red arrows), as well as many lymphocytic infiltrates (blue arrows) being observed. The aerial extract group showed a substantial increase in the number of viable neurons (red arrowheads), yet decreased red neurons (black arrows), attenuated perineuronal edema (black arrowheads), gliosis (red arrows) and lymphocytic infiltrates (blue arrows). The root extract group showed a mild increase in the number of viable neurons (red arrowheads). There are some red neurons (black arrows) and moderate perineuronal edema (black arrowheads), gliosis (red arrows) and lymphocytic infiltrates (blue arrows). (H&E, 200×. Scale bar = 50 µm).

Regarding histopathological examination for brain tissues, the normal group showed normal histopathological appearance, while the PTZ group showed obvious (grade 3) lesions involving 80% of the tissue section with many red neurons, perineuronal edema, gliosis and lymphocytic infiltrate. However, the aerial extract (400 mg/kg) group showed mild (grade 2) lesions involving 40% of the tissue section with few red neurons. Furthermore, the root extract (400 mg/kg) group showed moderate neuron lesions (grade 3) with 60% of the tissue section being involved (Figure 5).

**Figure 6:**
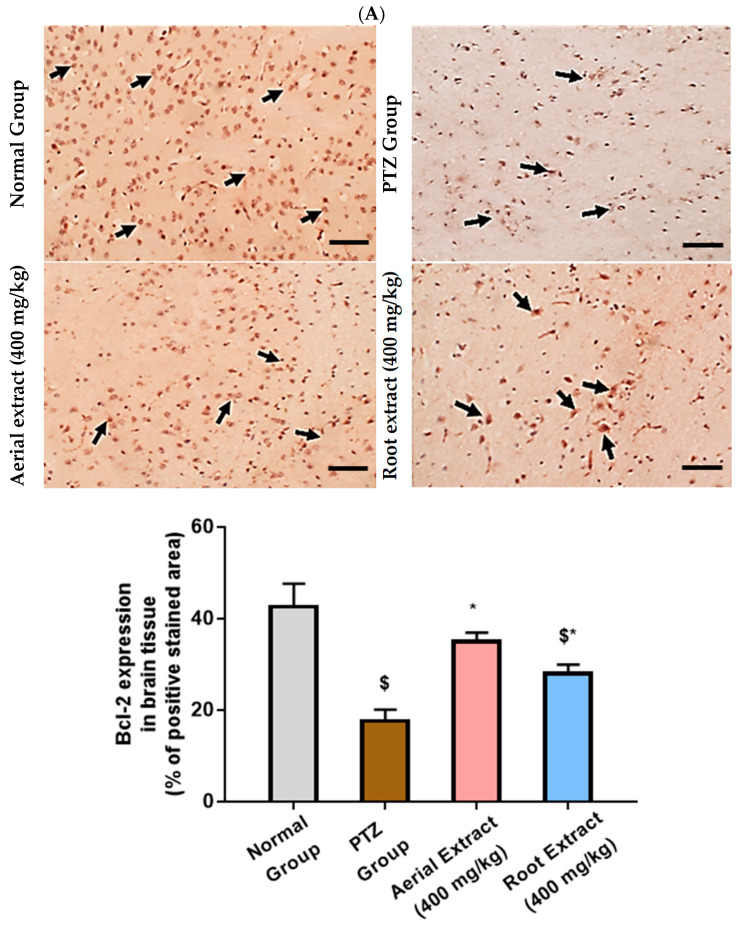

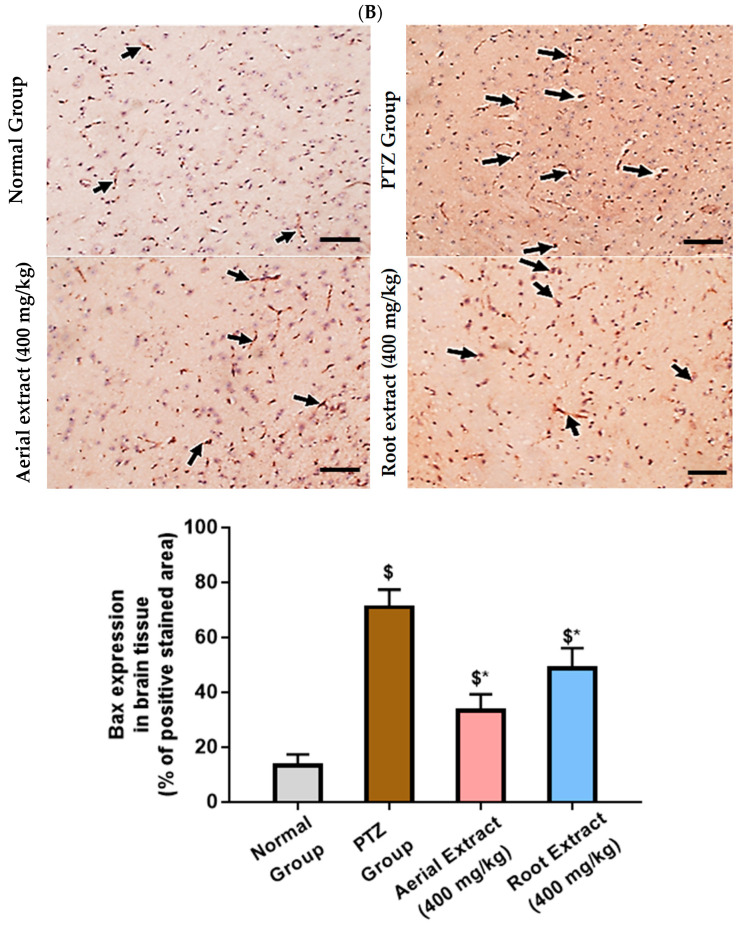
The effect of *Z. album* aerial parts and roots crude extracts on the immunoexpression levels of apoptotic markers in the brain tissue of the experimental mice. (**A**) Bcl-2 expression and (**B**) Bax expression. Positive immunofluorescence cytoplasmic reactions are indicated by black arrows. Bcl-2 = B cell lymphoma-2 and Bax = Bcl-2 associated x. Data of the percentage of positive stained area are expressed as mean ± SD. Analysis was performed using one-way ANOVA followed by Tukey’s post hoc test (*n* = 8). ^$^ significantly different vs. the normal control group and * significantly different vs. the PTZ group. Differences were considered significant at *p <* 0.05. (IHC, 200×. Scale bar = 50 µm).

Of note, Bcl-2 family members act as key regulators in the apoptotic process. The ratio of the pro-apoptotic Bax to the anti-apoptotic Bcl-2 determines survival or death of a cell following an apoptotic stimulus. It has been proposed that high Bax and/or low Bcl-2 as well as a high Bax/Bcl-2 ratio favors apoptosis [64]. As shown in Figure 6, the normal group showed strong Bcl-2 expression in many neurons, yet weak Bax expression. In the PTZ group, there is a significant reduction in neuronal Bcl-2 expression and an increase in Bax expression, pointing to a state of enhanced apoptosis. Such alterations of Bcl-2 and Bax immunoexpression were significantly reversed in both aerial and root extract groups, as compared to the PTZ group (*p* < 0.05). Additionally, Bcl-2 immunoexpression levels observed in the aerial extract group approached normal values.

Epileptic seizures have been documented to be associated with altered levels of excitatory and inhibitory neurotransmitter levels with neuronal hyper-excitability and deregulated neural connectivity [10,65]. Various factors such as excitotoxicity, mitochondrial dysfunction, oxidative stress and neuroinflammation have been reported to be implicated in epileptogenesis [65].

Given the multi-targeted effects of various natural compounds and their relative clinical safety, a rising interest has been paid to the use of antioxidant and anti-inflammatory natural products as respectable alternatives to control epileptogenesis and reduce the side effects of antiepileptic drugs [11]. Here, our aim was to evaluate the potential antiepileptic effect of *Z. album* aerial parts and roots crude extracts against PTZ induced kindling in mice.

PTZ, a gamma-aminobutyric acid (GABA) receptor antagonist, has been reported to induce epileptic seizures through the blockade of GABA receptors, which is a principal inhibitory neurotransmitter [66]. Chemical kindling can be achieved via repetitive PTZ injections at a subconvulsive dose (35 mg/kg, i.p.) administered on every alternate day to reach a total of 14 ± 1 injections [67]. In the same line, current results showed that repetitive i.p. injection of a subconvulsive dose of PTZ (35 mg/kg) on alternate days provoked kindling in PTZ-administered mice on the 15th PTZ injection with all experimental mice in the PTZ group exhibiting stage 4 and 5 seizures. The final seizure score (4.63 ± 0.52) exhibited by PTZ mice was significantly higher than that exhibited by the control mice (0 ± 0), *p* < 0.001. There was a gradual rise in seizure susceptibility during the course of kindling culminating in generalized tonic–clonic seizures. Furthermore, histological examination showed that the PTZ group showed obvious (grade 3) lesions involving 80% of the tissue section with many red neurons and substantial perineuronal edema, gliosis and lymphocytic infiltrates.

Interestingly, treatment with *Z. album* aerial parts and roots crude extracts significantly decreased the final seizure score and the percentage of fully kindled mice (25% and 62.5%, respectively) compared to the PTZ group. No mice recorded stage 5 seizures in either treatment groups. Such effect was further confirmed with histopathological examination of brain tissues which revealed that the aerial extract group showed mild (grade 2) lesions involving 40% of the tissue section with few red neurons, attenuated perineuronal edema, gliosis and lymphocytic infiltrates. Furthermore, the root extract group showed moderate neuron lesions (grade 3) with 60% of the tissue section being involved. It is noteworthy mentioning that the effect of the aerial extract on the final seizure score, as well as on PTZ-induced histopathological changes, was superior to that of the root extract.

Of note, reactive free-radical generation and oxidative stress can considerably alter neuronal function and contribute to the progression of epileptogenesis and neuronal death. Previous studies demonstrated that the PTZ-induced kindling model was associated with impaired antioxidant mechanisms, with the activities of the antioxidant enzymes SOD and catalase being declined in the brain of PTZ-kindled mice [65,66]. Impaired antioxidant defense has been reported to trigger mitochondrial injury with subsequent elevated production of reactive oxygen and nitrogen species. As a result, PTZ injection enhances lipid peroxidation and increases MDA levels, together with elevated NO levels that could be attributed to iNOS activation with the subsequent peroxynitrite radicals formation [68]. Similarly, data from this study revealed increased oxidative stress in the PTZ group as evinced by significantly elevated MDA levels and iNOS activity, parallel to decreased TAC and SOD and catalase activities, as compared to the normal group.

*Z. album* aerial parts and roots crude extracts had the ability to reverse oxidative damage caused by PTZ. Significant decline in iNOS and MDA levels, as well as a significant increase of the total antioxidant capacity, SOD and catalase levels in the brain tissue, were observed in mice pre-treated with *Z. album* aerial parts and roots crude extracts relative to the untreated mice. Moreover, the group that received the *Z. album* aerial parts extract showed the greatest improvement in these parameters. In agreement with these results, pretreatment with *Z. album* lowers MDA and raises catalase and SOD activity, which helps to mitigate the oxidative stress in mice’s testicular injury caused by methotrexate [6].

A growing body of evidence has shown that inflammatory processes could mediate neuronal cell death and exacerbate neuronal excitability consequently participating in the etiology and clinical progression of epileptogenesis [10]. NLRP3, the most classic inflammasome, is implicated in a wide range of pathologies including epilepsy with NLRP3 being the sensor protein, ASC being the adaptor protein and caspase-1 being the effector protein. It is activated by binding of TNF or IL-1β to different types of pattern recognition receptors (PRRs) including toll-like receptors (TLRs) and nucleotide-binding oligomerization domain (NOD), leucine-rich repeat (LRR)-containing protein receptors (NLRs) and cytokine receptors [69]. Such activation leads to enhanced transcription of NLRP3 and pro-IL-1β, oligomerization and auto-proteolytic maturation of pro-caspase-1 which cleaves pro-IL-1β and pro-IL-18 into their active forms [69]. In the same context, TXNIP links redox signaling with inflammasome activation as it dissociates from Trx in a reactive oxygen species (ROS)-sensitive manner and is allowed it to bind NLRP3 with subsequent inflammasome activation [11,70].

Indeed, activated NLRP3 inflammasome with subsequent endorsed release of inflammatory cytokines has been previously reported in the hippocampus with PTZ-induced kindled mice [10]. Current results were consistent with those of previous findings where PTZ-kindled mice experienced activated NLRP3 inflammasome and revealed significantly upregulated gene expression of *NLRP3*, *TXNIP* and *caspase-1*, parallel to elevated IL-1β and IL-18 levels, as compared to the normal group.

According to previous reports, *Z. album* extract lowers proinflammatory cytokines and inflammation [6]. This is consistent with the current findings, which show that, when compared to the PTZ group, the groups pretreated with either the aerial part or the root extract of *Z. album* showed a significant inhibition in the NLRP3 inflammasome pathway and significantly downregulated *NLRP3*, *TXNIP* and *caspase-1* expression, along with reduced levels of IL-1β and IL-18. Indeed, NLRP3 inflammasome inactivation has been reported to be a potential mechanism of action of various compounds found in *Z. album* extract [71,72,73,74,75]. A better outcome has been observed with aerial part extract than the root extract.

Importantly, oxidative stress and neuroinflammation have been demonstrated to aggravate apoptosis of neurons in epilepsy [10,65]. Rong et al. [10] have reported a significantly decreased Bcl-2/Bax ratio and increased expression of cleaved caspase-3 following PTZ-induced kindling. Here, the PTZ group showed significantly decreased Bcl-2 immunopositivity yet an increased number of Bax positive cells in the hippocampus as compared to normal group.

Moreover, current results revealed that administering *Z. album* extract reversed PTZ-induced altered Bcl-2 and Bax expression, pointing to the fact that the *Z. album* neuroprotective effect could be attributed to inhibition of apoptosis with the antiapoptotic effect of the aerial part being superior to that of the root extract. A similar result by Feriani et al. [76] showed the antiapoptotic effects of *Z. album* leaf extract in a rat model of deltamethrin-induced hepatic fibrosis by virtue of its phytochemical composition.

## 4. Conclusions

Metabolomic profiling of *Z. album* extracts exhibited a number of secondary metabolites including flavonoids, triterpenes and phenolic compounds. To the best of our knowledge, this is the first study evaluating the anti-epileptic effect of *Z. album* aerial parts and roots crude extracts in a PTZ-induced kindling model. Current findings revealed that *Z. album* aerial parts and roots crude extracts attenuated seizure severity score in kindled mice and improved histological changes in the brain accompanying epileptogenesis. Such neuroprotective effects could be attributed to decreased oxidative stress as evinced by reduced iNOS and MDA levels, as well as restored total antioxidant capacity and levels of antioxidant enzymes (SOD and catalase). Moreover, *Z. album* aerial parts and roots crude extracts inhibited PTZ-induced activation of NLRP3 inflammasome with the expression of the inflammatory cytokines (IL-1β and IL-18) being decreased. Furthermore, administration of *Z. album* augmented Bcl-2 yet decreased Bax immunoexpression in the kindled mice brain with subsequent inhibition of apoptosis. Interestingly, the antiepileptic effect of the *Z. album* aerial parts extract was superior to that of the roots which was associated with higher antioxidant, anti-inflammatory and antiapoptotic activity. We hypothesize that the effects described above are due to the higher amount of polyphenols present in the aerial parts than in the roots. However, further studies are required to determine whether this difference is due to different polyphenol content or to compounds present only in the aerial extract.

## Figures and Tables

**Table 1:** Annealing temperature and primer sequences for the assessed genes.

GenBank Accession No.	Gene	Primers	Annealing Temperature	Reference
NM_145827.4	*NLRP3*	Forward: 5′-AGCCTTCCAGGATCCTCTTC-3′	52 °C	[26]
		Reverse: 5′-CTTGGGCAGCAGTTTCTTTC-3′		
NM_001009935.2	*TXNIP*	Forward: 5′-GATACCCCAGAAGCTCCTCC-3′	54 °C	[27]
		Reverse: 5′-ACCTCAGTGTAAGTGGGTGG-3′		
NM_009807.2	*Caspase-1*	Forward: 5′-TGGCAGGAATTCTGGAGCTT-3′	53 °C	[28]
		Reverse: 5′-CTTGAGGGTCCCAGTCAGTC-3′		
NM_001289726.2	*GAPDH*	Forward: 5′-ATGACTCTACCCACGGCAAG-3′	55 °C	[29]
		Reverse: 5′-GATCTCGCTCCTGGAAGATG-3′		

## Data Availability

The data presented in this study are available in the main article and the Supplementary Materials.

## Supplementary Materials

The following supporting information can be downloaded at: https://www.mdpi.com/article/10.3390/metabo14060316/s1, Figure S1: Aerial parts crude extract: Negative-MODE–TIC; Figure S2. Aerial parts crude extract: Negative-MODE–BPC; Figure S3. Roots crude extract: Negative-MODE–TIC; Figure S4. Roots crude extract: Negative-MODE–BPC; Figure S5. Aerial parts crude extract: Positive-MODE–TIC; Figure S6. Aerial parts crude extract: Positive-MODE–BPC; Figure S7. Roots crude extract: Positive-MODE–TIC; Figure S8. Roots crude extract: Positive-MODE–BPC.
