# Supplementary material for: Metabolomic Profiling and In Vivo Antiepileptic Effect of Zygophyllum album Aerial Parts and Roots Crude Extracts against Pentylenetetrazole-Induced Kindling in Mice
Source: Metabolites. 2024 May 30;14(6):316. doi: 10.3390/metabo14060316 (PMC11205424; doi:10.3390/metabo14060316)
Supplement: Supplementary file 1 [file metabolites-14-00316-s001.zip › metabolites-2971179-supplementary.pdf]

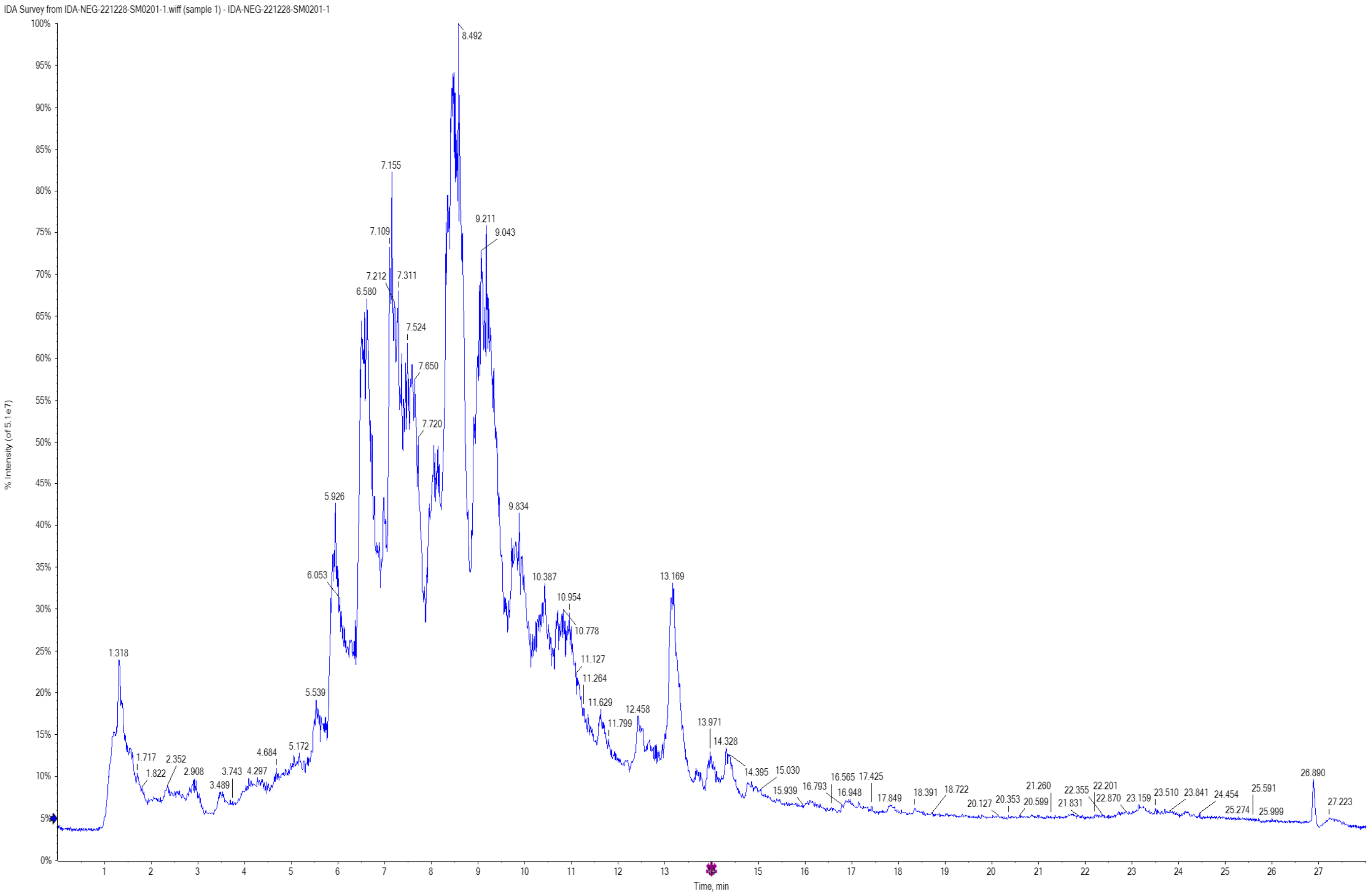

FigureS1. Aerial parts crude extract: Negative -MODE - TIC

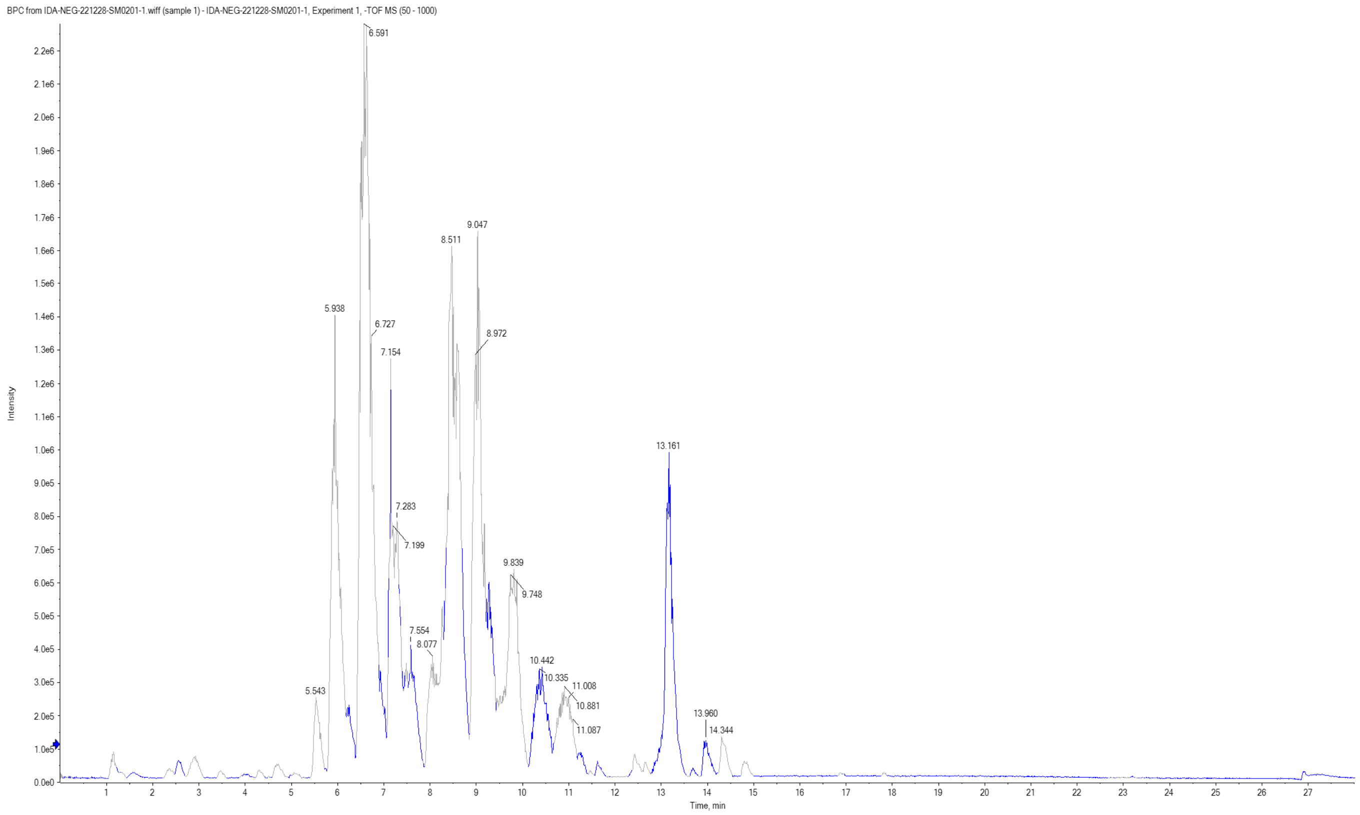

Figure S2. Aerial parts crude extract: Negative -MODE - BPC

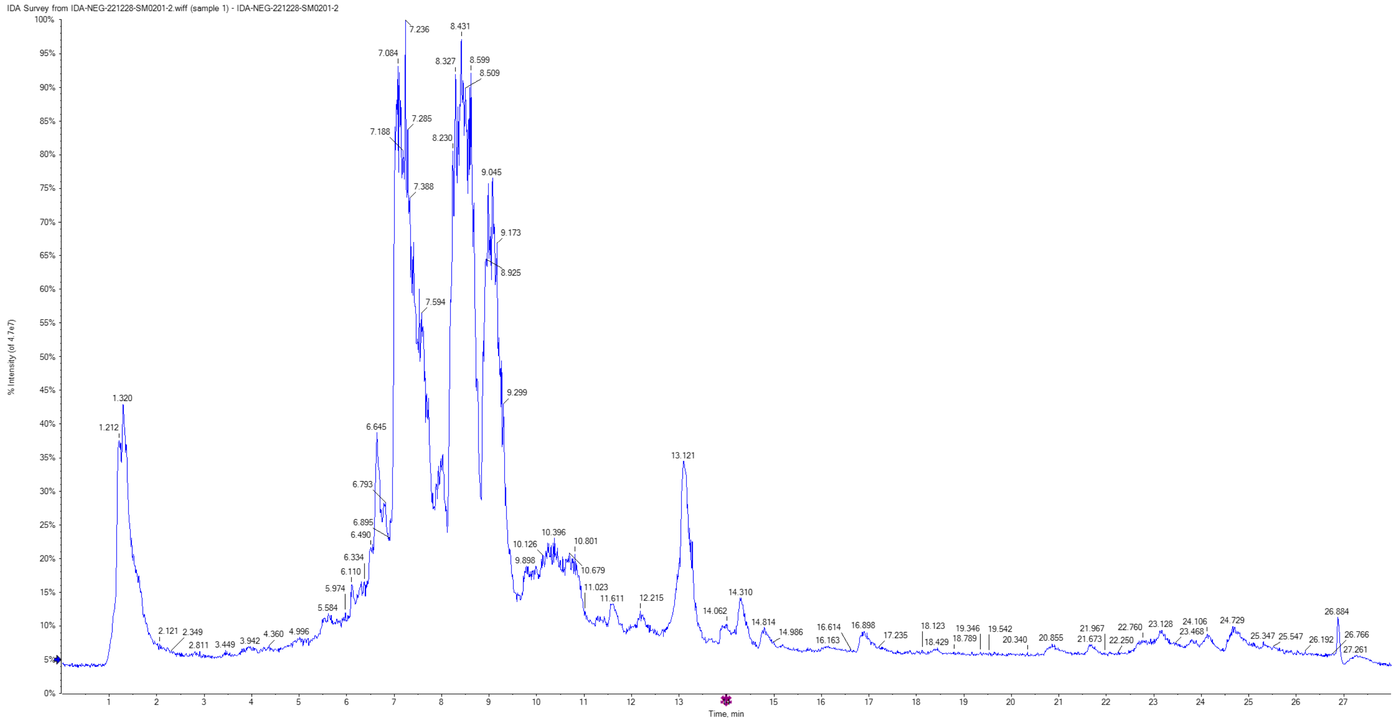

Figure S3. Roots crude extract: Negative -MODE - TIC

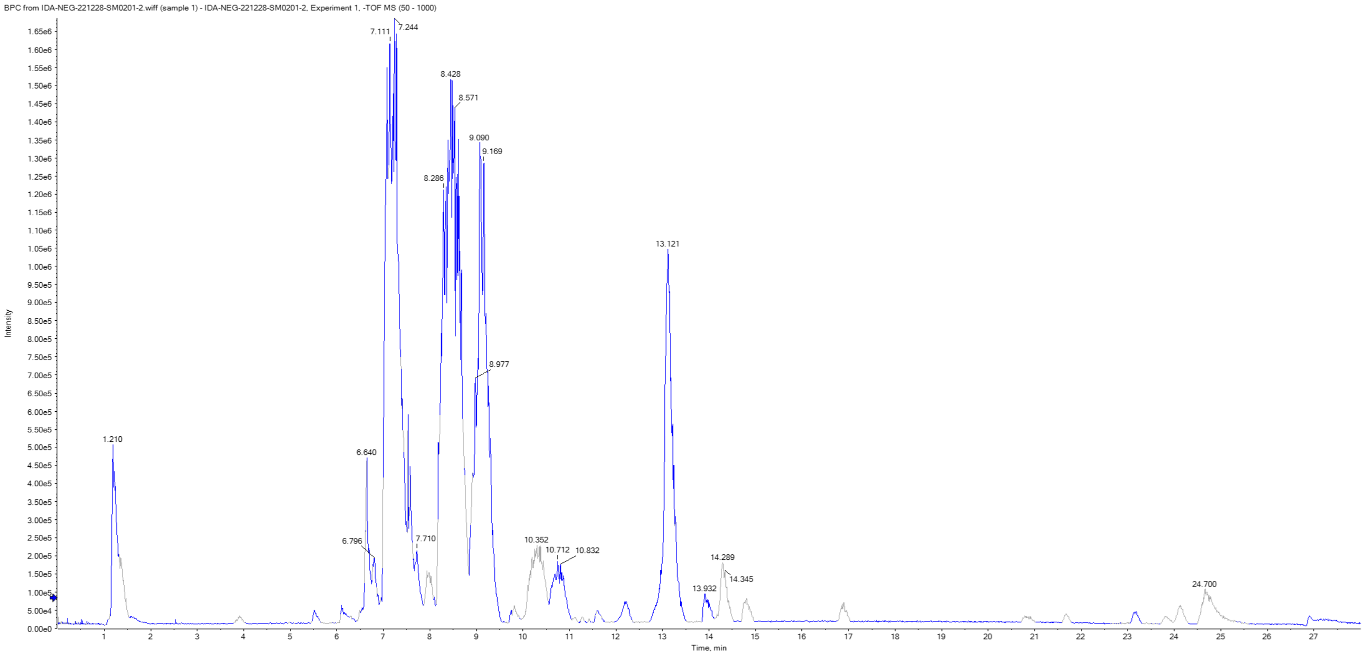

Figure S4. Roots crude extract: Negative -MODE - BPC

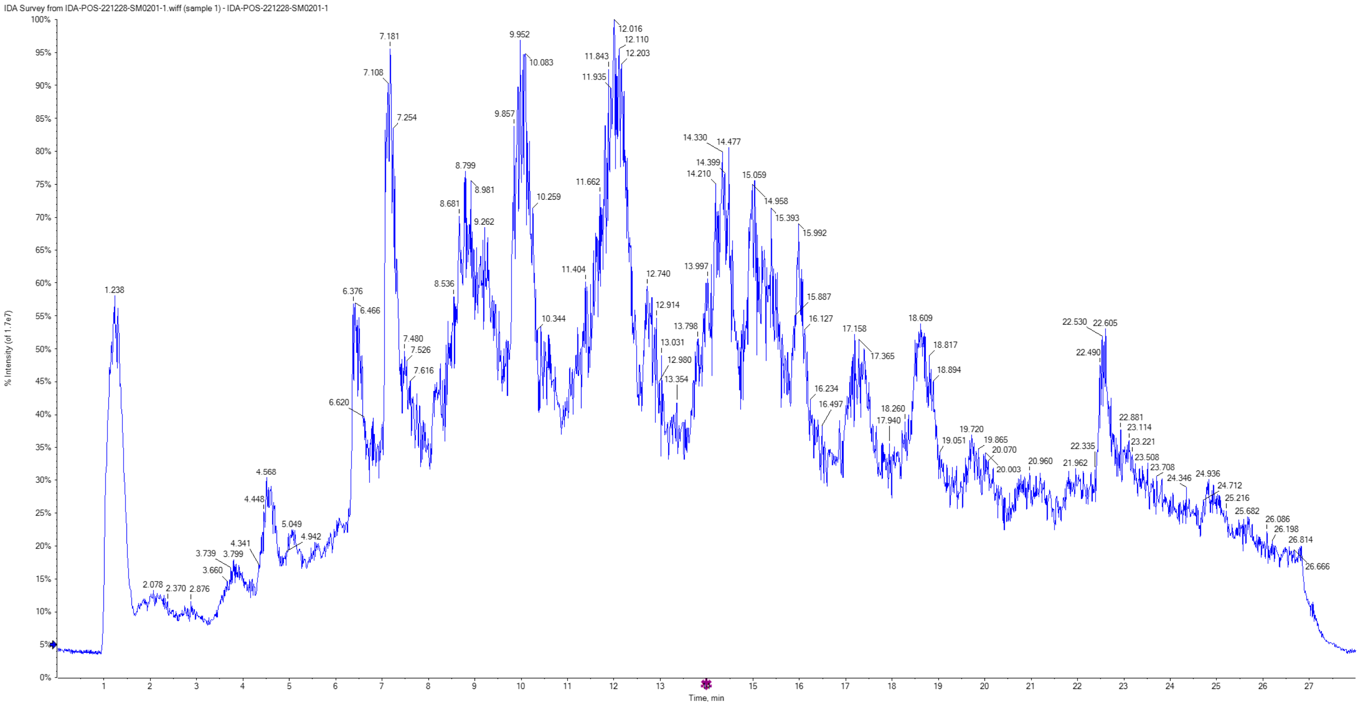

Figure S5. Aerial parts crude extract: Positive -MODE - TIC

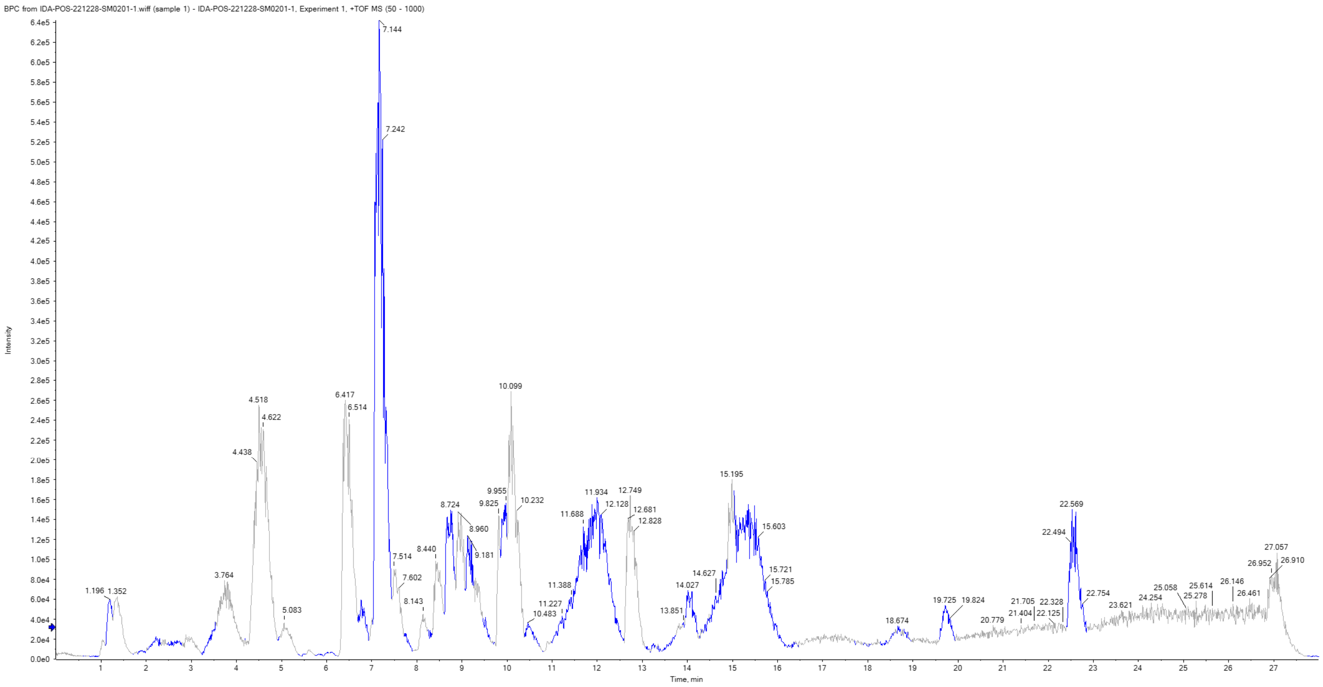

Figure S6. Aerial parts crude extract: Positive -MODE – BPC

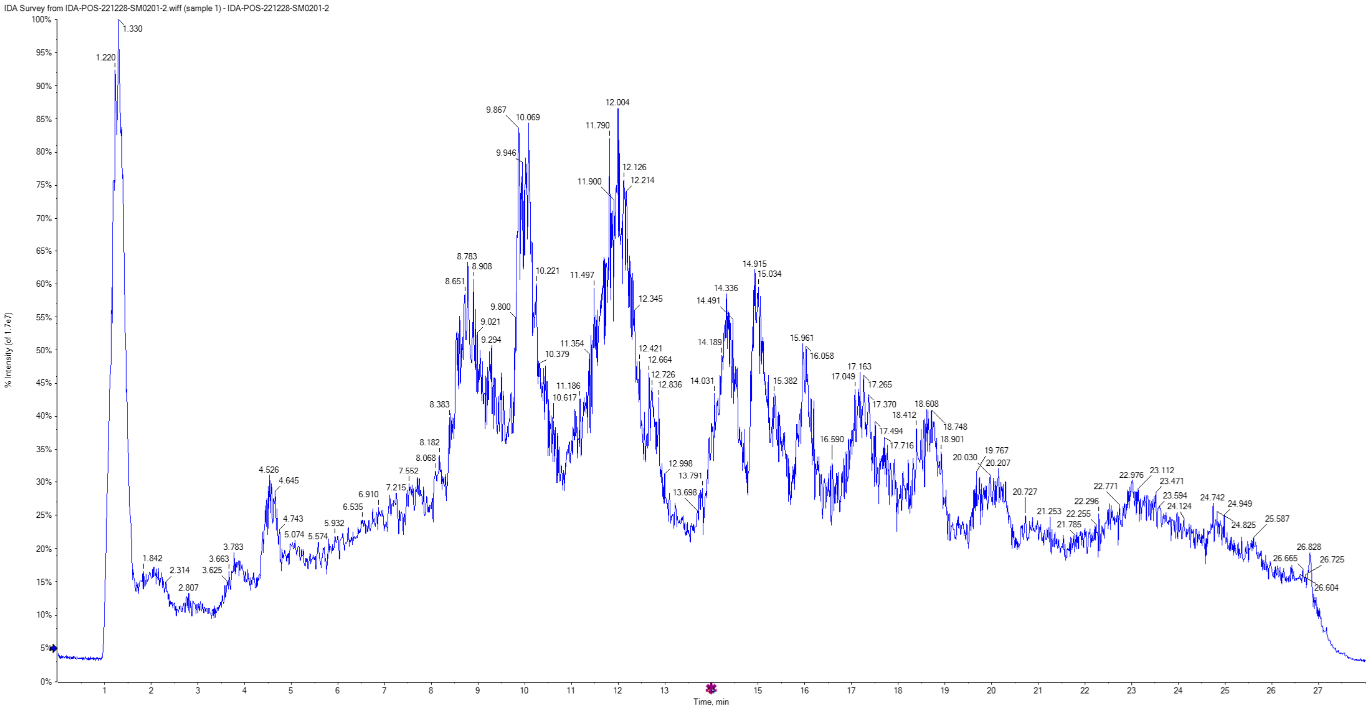

Figure S7. Roots crude extract: Positive -MODE – TIC

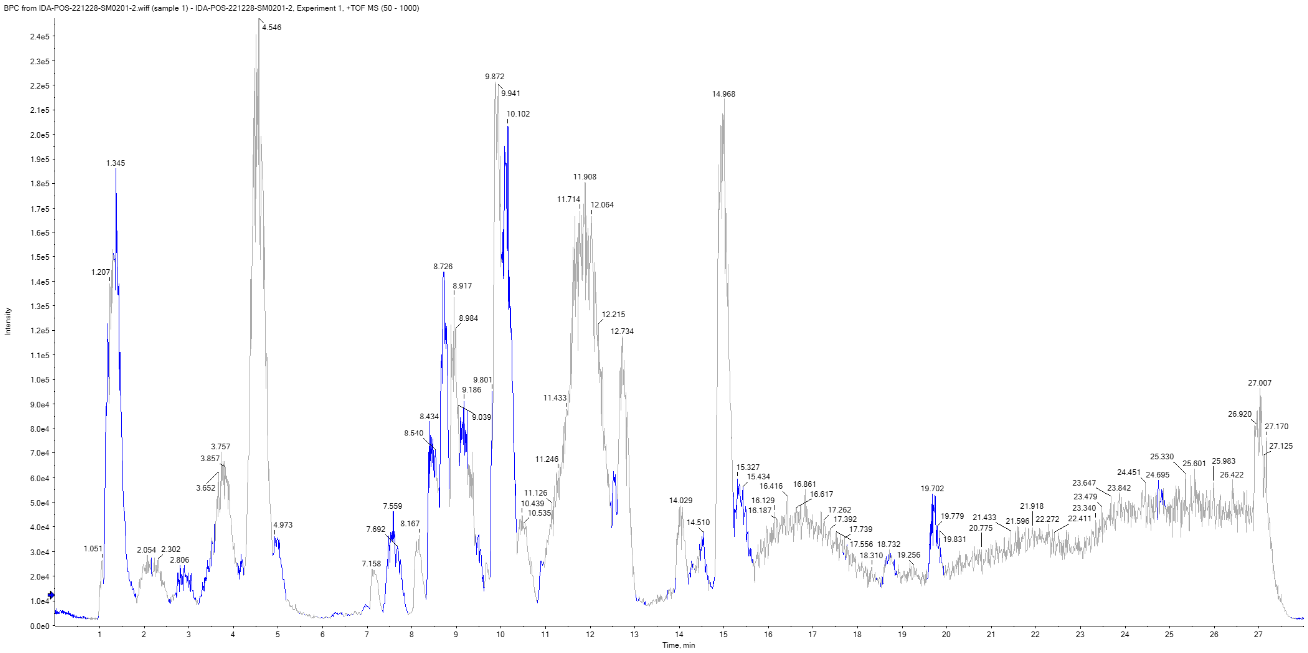

Figure S8. Roots crude extract: Positive -MODE - BPC
